# Identification and Characterization of Phospholipase D Genes Putatively Involved in Internal Browning of Pineapple during Postharvest Storage

**DOI:** 10.3389/fpls.2017.00913

**Published:** 2017-06-19

**Authors:** Keqian Hong, Lubin Zhang, Rulin Zhan, Bingyu Huang, Kanghua Song, Zhiwei Jia

**Affiliations:** Key Laboratory for Postharvest Physiology and Technology of Tropical Horticultural Products of Hainan Province and Key Laboratory of Tropical Fruit Biology, Ministry of Agriculture, South Subtropical Crop Research Institute, Chinese Academy of Tropical Agricultural SciencesZhanjiang, China

**Keywords:** pineapple fruit, phospholipase D, internal browning, gene expression, defense response

## Abstract

Phospholipase D (PLD) in plants plays vital roles in growth, development, and stress responses. However, the precise role of PLDs in pineapple remains poorly understood. In this study, 10 putative *PLD* genes, designated as *AcPLD1*–*AcPLD10*, were identified based on the pineapple genome database. The 10 AcPLDs could be clustered into five of the six known PLD families according to sequence characterization. Their deduced amino acid sequences displayed similarities to PLDs from other plant species. Expression analyses of *PLD* mRNAs from pineapple pulp were performed. The 10 *PLDs* exhibited differential expression patterns during storage periods of fruits treated with hexaldehyde (a specific PLD inhibitor) which could alleviate internal browning (IB) of pineapple after harvest. Functional subcellular localization signaling assays of two PLD proteins (AcPLD2 and AcPLD9) were performed by fluorescence microscopy. To further detect the potential action mechanism underlying PLD involved in the IB defense response, PLD, hydrogen peroxide (H_2_O_2_) and H_2_O_2_ associated with antioxidative enzymes such as superoxide dismutase, catalase, NADPH, and ascorbate peroxidase were quantified by enzyme-linked immunosorbent assay. This report is the first to provide a genome-wide description of the pineapple *PLD* gene family, and the results should expand knowledge of this family.

## Introduction

Phospholipase D (PLD, EC 3.1.4.4) which hydrolyzes different membrane phospholipids (PLs) into phosphatidic acid and various water-soluble head groups, such as choline or ethanolamine, has been detected in bacteria, fungi, plants, and animals (Guo and Wang, 2012; Comoglio et al., 2014). Since plant PLD cDNA was first cloned by Wang et al. (1994), many PLD-encoding genes have been obtained from plants, such as rice (Ueki et al., 1995), *Arabidopsis thaliana* (Pinosa et al., 2013), tobacco (Lein and Saalbach, 2001), tomato (Whitaker et al., 2001), strawberry (Yuan et al., 2005), and soybean (Zhao et al., 2012). Protein structural analysis has resulted in several conserved domains identified in PLDs, including PLD-C1 and PLD-C2 domains, C2 domain, and PX (phox)/PH (pleckstrin homology) domains. PLD-C1 and PLD-C2 domains are also called HKD domains, which are regarded as catalytic sites and are essential for the catalytic activity of the PLD enzyme (Qin and Wang, 2002). The C2 domain, is known as the Ca^2+^/PL-binding domain, which can mediate Ca^2+^-dependent activity through binding to Ca^2+^ (Kopka et al., 1998). PX/PH domains, which are located at the N-terminus of Ca^2+^-independent PLDs (Kopka et al., 1998; Qin and Wang, 2002), have been implicated in membrane targeting and polyphosphoinositide signaling of PLD (van Leeuwen et al., 2004).

An increasing number of studies have indicated that PLD, which is considered an important signaling enzyme in various organisms and is widespread in plants (Jang et al., 2012), participates in diverse physiological processes, such as development (Lu et al., 2016); hormone signaling mediated by abscisic acid (Peters et al., 2010), salicylic acid (Janda et al., 2015), jasmonic acid (Zhao et al., 2013), and auxin (Li and Xue, 2007); reactive oxygen generation (Wu et al., 2010); and in the response to abiotic and biotic stresses such as drought (Lu et al., 2012), high salinity (Yu et al., 2010), low temperature (Kargiotidou et al., 2010), and fungal infection (Liu et al., 2013). The diverse cellular functions of PLD suggest that the enzyme is subjected to complex regulation in the cell.

Although the functions of PLD in many plants have been studied extensively, the specific role of different plant PLDs remains unknown. Moreover, the involvement of PLDs in relation to quality of fruits after harvest has received minimal attention. Pineapple (*Ananas comosus* L.), which is the second most important harvest crop after banana and tasty tropical fruit, is cultivated in over 80 countries worldwide and produced annually with a gross production value approaching nine billion dollars (FAO, 2014). China produced approximately 1.89 million tons of pineapple in 2014, accounting for 7.4% of worldwide production (FAO, 2014). Under natural conditions, pineapples undergo internal browning (IB) during postharvest storage (Zhang et al., 2015). Therefore, the quality and storage potential of this fruit must be improved. Effective methods to delay the development of IB include 1-methylcyclopropene treatment (Selvarajah et al., 2001), controlled atmosphere (Nimitkeatkai et al., 2006), waxing (Hu et al., 2012), and exogenous abscisic acid (Zhang et al., 2015), but the mechanism of the IB remains unclear. The recent completion of the pineapple genome has allowed for the genome-wide identification of a number of important gene families (Ming et al., 2015). Three different PLD inhibitors, namely, 1-butanol (Pinosa et al., 2013), lysophosphatidyl ethanolamine (Li et al., 2015), and hexaldehyde (Paliyath et al., 2003), can all delay the development of IB in pineapple after harvest. Thus, PLDs may be the involved in IB. However, the functions of PLDs in pineapple need to be elucidated. This study was conducted to address the detailed structural analyses of PLDs in pineapple, as well as determine their phylogenetic relationship with other species. The potential regulatory mechanisms of PLD involvement during IB in pineapple are also discussed.

## Materials and Methods

### Plant Materials and Treatments

Pineapple (*A. comosus* cv. ‘Comte de Paris’) at stage 3 of maturation as proposed by Selvarajah et al. (2001) was obtained from a commercial orchard in Xuwen County, Guangdong Province in winter. Fruits of uniform size, color, and maturity, as well as being free of physical injury or disease symptoms, were selected, with the crown intact. Subsequently, fruits were immersed for 2 min in 0.05% (w/v) benomyl aqueous solution to control decay, air-dried at 25°C for 2 h and treated as follows. The selected pineapple fruits were immersed into 0.1% (v/v) hexaldehyde containing 0.01% Tween 80 for 5 min. The control fruits were immersed with distilled water, which also contained 0.01% Tween 80. Each treatment was applied to three replications, and each replication contained 100 fruits. Following treatment, all fruits were placed into unsealed plastic bags (0.04 mm thick), and stored at 25°C in the dark with relative humidity at 95% for 12 days. Samples of pulp tissues were collected at days 0, 2, 4, 6, 8, 10, and 12 frozen in liquid nitrogen; and stored at -80°C for future use.

### Evaluation of IB Intensity

For IB assessment, at least six fruits for each treatment were halved longitudinally to observe the incidence and severity of IB injury. IB intensity was visually scored from 0 (no IB) to 5 (maximum IB) according to Teisson (1979).

### Identification of PLD and Sequence Analysis

A putative pineapple PLD cDNA was identified by searching the pineapple genome database. The homology of predicted PLD protein sequences was checked using Clustal W, with the default parameters set by the DNA Data Bank of Japan^1^. Domain and motif searches were conducted on the protein sequences in SMART^2^. Blast searches were also performed against nucleic acid sequence data repositories at the National Center for Biotechnology Information (NCBI^3^). A phylogenetic tree was generated using Clustal Omega online tools^4^. The logos of the conserved domains were constructed using MEME program^5^ with the default parameters. The theoretical isoelectric point (pI) and molecular weight of the pineapple PLD proteins were calculated using PeptideMass program^6^. Transmembrane helix number was predicted by HMMTOP online tools^7^.

### Quantitative Real-Time PCR (qRT-PCR) Analysis

Total RNA was extracted from pineapple pulp using the hot borate method of Wan and Wilkin (1994). RNA purification and first-strand cDNA synthesis were performed as described in our previous paper (Hong et al., 2016). qRT-PCR analyses were conducted to detect gene expression patterns of the PLD family during the development of IB in fruits treated with PLD inhibitor (hexaldehyde). The oligonucleotide primers for qRT-PCR analysis were designed using Primer 5.0 software and *Acactin* (accession number HQ148720) was used as a reference gene. Reactions were carried out using SYBR^^®^^ premix Ex Taq^TM^II (Takara, Shiga, Japan) on a LightCycler^^®^^480 II (Roche, Switzerland). The relative expression levels of *AcPLDs* were calculated using the method of Livak and Schmittgen (2001). Values represented the average of three biological replicate. The sequences of all primers are listed in Supplemental File 1.

### Subcellular Localization Analysis

The coding sequences of AcPLD2 and AcPLD9 without the stop codon were amplified by PCR (primers are listed in Supplemental File 2) and subcloned into the pEAQ-GFP-HT vector to form fusion constructs AcPLD2::GFP and AcPLD9::GFP, under the control of the CaMV 35S promoter. The fusion constructs and a control vector were maintained in *Agrobacterium tumefaciens* strain EHA105 by electroporation. Transient transformation of tobacco leaves was carried out according to the method described by Sainsbury et al. (2009). Localizations of AcPLD2::GFP and AcPLD9::GFP were investigated with a fluorescence microscope (Zeiss Axioskop 2 Plus). All transient expression assays were repeated three times.

### Measurements of Superoxide Dismutase (SOD), Catalase (CAT), NADPH, Ascorbate Peroxidase (APX), PLD, and Hydrogen Peroxide (H_2_O_2_) Contents

The pineapple pulp samples (1.0 g) were homogenized in 9.0 ml of phosphate buffered saline (PBS) (pH 7.4) and then centrifuged at 4000 ×*g* for 15 min. The supernatant solution was collected for analyses of SOD, CAT, NADPH, APX, PLD, and H_2_O_2_ contents, which were quantified by enzyme-linked immunosorbent assay (ELISA). ELISA was carried out as follows: (1) The 96-well microplates were coated with 100 μL of the captured specific antibody at 1:2000 dilution in coating buffer and incubated overnight at 4°C. (2) Plates were washed three times with PBS to remove unbound antibody, and each well was filled with 300 μL of blocking buffer and incubated for 30 min at 37°C. (3) Followed by washing, 100 μL sample solution mentioned above was added to each well and incubated for 30 min at 37°C. (4) After washing three times, 100 μL horseradish peroxidase-conjugated the specific antibody was added to each well and incubated at 37°C for 30 min. (5) The plate was washed again, and 100 μL of solution 3,3/5,5/-tetramethylbenzidine was added to each well. (6) After developing the color for 30 min at 37°C, the reaction was terminated after the addition of 50 μL of 2 M H_2_SO_4_ per well. (7) The absorbance values at 450 nm were determined, and the values of ELISA were given in relative units (ng.mg^-1^protein). Protein content was measured by the method of Bradford (1976) using bovine serum albumin as the standard protein.

### Statistical Analysis

The experiment was performed in triplicate (*n* = 3). Data were presented as the mean ± standard error. Least significant difference at the 5% level was analyzed by SPSS software (version 13.0, SPSS Inc., Chicago, IL, United States).

## Results

### IB Intensity

**FIGURE 1:**
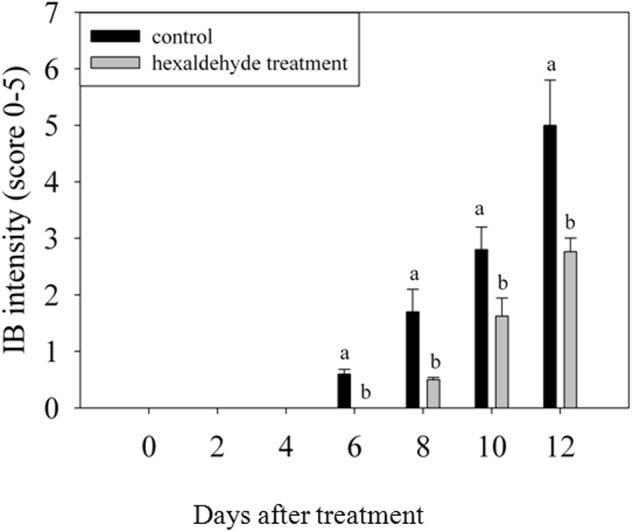
Changes in IB intensity of fruits treated with hexaldehyde. Each value represents the mean ± standard error of three replicates.

Control fruits showed signs of IB after 6 days of storage, whereas no sign of IB was detected in fruits treated with hexaldehyde. When the treated fruits began to exhibit the IB at day 8 of storage, the IB intensity reached 1.7 in control samples. IB progressively increased as storage duration increased. The IB intensity was higher for control samples than for treated fruits, and a noticeable difference was observed between them after 6 days of storage (**Figure 1**).

### Identification of *PLD* Gene Family Members in Pineapple

**FIGURE 2:**
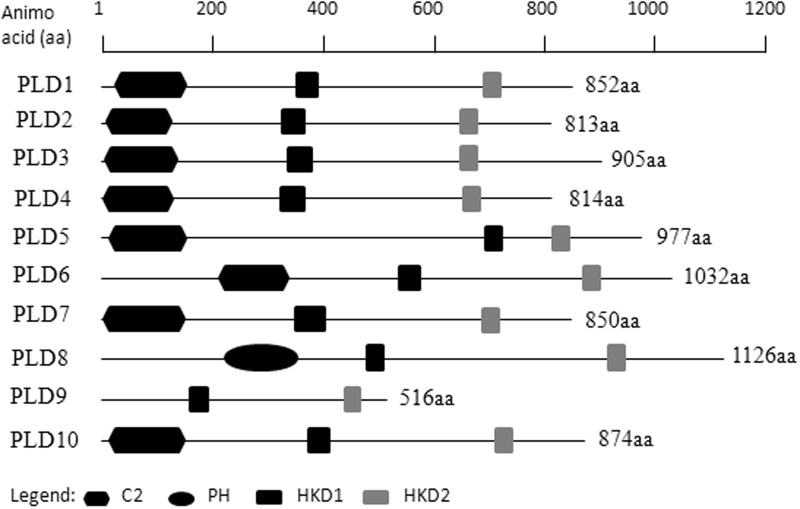
Structural comparisons of pineapple PLDs domains. Protein structures and domains of pineapple PLD family members PLD1-10. Top line represents amino acid (aa) position. C2, protein kinase C-conserved 2 domain; PH, pleckstrin homology domain; HKD1, HKD1 domain; HKD2, HKD2 domain.

**FIGURE 3:**
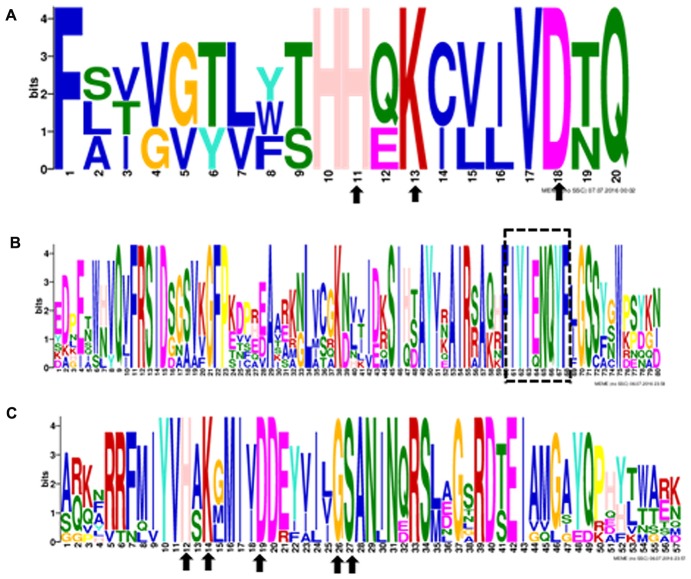
The conserved domains in 10 pineapple PLD proteins. Numbers on the *x*-axis represents the sequence compositions of each domain. The *y*-axis represents the information content measured in bits. **(A)** Alignment of the HKD1 domain. **(B)** Alignment of the “IYIENQYF” domain. **(C)** Alignment oftheHKD2 domain.

Ten putative *PLD* genes were identified in pineapples by searching against the pineapple genome database. These genes were located on chromosomes 1, 2, 3, 5, 7, 8, 11, 18, 19, and 25, and their temporary names were designated as *AcPLD1*–*AcPLD10* based on their order on the pineapple chromosome from 1 to 25 (**Table 1**). *AcPLD1*–*AcPLD10* were predicted to encode proteins of 852, 813, 905, 814, 977, 1032, 850, 1126, 516, and 874 amino acids, respectively, with calculated molecular weights of 96.6, 91.2, 101.7, 92.4, 111.0, 114.5, 96.3, 127.8, 57.3, and 98.7 kDa, respectively, and pI values of 8.00, 5.88, 6.57, 5.80, 6.73, 6.91, 6.78, 6.50, 7.15, and 6.79, respectively. Alignment of the full-length deduced proteins of the AcPLDs clearly showed that the proteins contained two conserved HKD domains (HKD1 and HKD2) in their sequences. HKD1 regions displayed more diversity than HKD2 regions (**Figures 2**, **3**). The two HKD domains were separated by 320 amino acids, except for PLD3, PLD5, PLD8, and PLD9, which demonstrated longer or shorter spacing sequences. The “IYIENQYF” motif, which was found among the 10 pineapple PLDs, with the exception of PLD9, was the most conserved domain with the least diversity. The C2 domain was present in pineapple PLDs, except PLD8 and PLD9. The PH domain was present in pineapple PLD8 but not in other pineapple PLDs (**Figures 2**, **3** and **Tables 1**, **2**). Alignment analysis indicated that the similarities of the deduced amino acids of AcPLDs varied from 10% (AcPLD4 and AcPLD9; AcPLD8, and AcPLD9) to 73% (AcPLD5 and AcPLD10; **Table 3**).

**Table 1:** The information of *AcPLD* gene family.

Name	Gene ID	Chr.	Gene locus	ORF (AA)	HKD1//HKD2	Transmembrane helix number
*PLD1*	Aco018042	1	20111511-20124381	852	+/320aa/+	0
*PLD2*	Aco016943	2	1792303-1797314	813	+/305aa/+	1
*PLD3*	Aco011986	3	14132196-14147669	905	+/387aa/+	0
*PLD4*	Aco014266	5	108322-114777	814	+/310aa/+	0
*PLD5*	Aco019548	7	11601803-11612907	977	+/105aa/+	1
*PLD6*	Aco013911	8	9807533-9814878	1032	+/315aa/+	1
*PLD7*	Aco016554	11	37825-52472	850	+/320aa/+	0
*PLD8*	Aco001838	18	7525795-7540516	1126	+/419aa/+	2
*PLD9*	Aco008209	19	8526583-8530850	516	+/262aa/+	0
*PLD10*	Aco013079	25	818670-824553	874	+/322aa/+	1

**Table 2:** The structural comparisons of AcPLD proteins containing HKD1, ‘IYIENQYF,’ and HKD2 domains.

Name	HKD1	IYIENQYF	HKD2
PLD1	**H**Q**K**CVIM**D**TQAGGNNRKI	IYIENQYF	**H**S**K**GMIV**D**DEYVII**GS**A**N**
PLD2	**H**Q**K**TVTL**D**APASD—**GS**SS	IYIQNQYF	**H**A**K**LMIV**D**DEYVIV**GS**A**N**
PLD3	**H**Q**K**CVIV**D**TQLDTQL**S**QS	IYIENQYF	**H**A**K**GMII**D**DEYVIL**GS**A**N**
PLD4	**H**Q**K**IVVV**D**HEMPNE**GS**QQ	IYIENQYF	**H**A**K**MMIV**D**DEYIIV**GS**A**N**
PLD5	**H**Q**K**CLLV**D**TQASGNDRKI	IYIENQYF	**H**A**K**GMIV**D**DEYVIL**GS**A**N**
PLD6	**H**Q**K**TVIM**D**ADAGH**G**RRK	IYIENQYF	**H**S**K**GMIV**D**DEFVIL**GS**A**N**
PLD7	**H**Q**K**TVIV**D**YEVPAA**G**NKK	IYIENQYF	**H**A**K**MMIV**D**DEYIII**GS**A**N**
PLD8	**H**E**K**IVIV**D**NQVCYI**GG**LD	IYIENQFF	**H**S**K**LMII**D**DRIALV**GS**A**N**
PLD9	**H**A**K**VWIS**D**RNSLYI**GS**A**N**	— — — —	**H**G**K**YAVS**D**VRAHI**GT**S**N**
PLD10	**H**Q**K**CLLV**D**TQASRNNRKI	IYIENQYF	**H**A**K**GMII**D**DEYVIL**GS**A**N**

**Table 3:** Amino acid sequence comparison between the predicted the full-length phospholipase D (PLD) cDNAs.

Amino acid similarity (%)									
	**PLD2**	**PLD3**	**PLD4**	**PLD5**	**PLD6**	**PLD7**	**PLD8**	**PLD9**	**PLD10**
PLD1	36	53	40	59	52	35	13	12	64
PLD2		35	48	37	39	45	14	11	33
PLD3			37	49	39	32	14	12	57
PLD4				41	42	68	14	10	35
PLD5					43	36	12	11	73
PLD6						36	12	11	43
PLD7							14	11	37
PLD8								10	14
PLD9									11

**FIGURE 4:**
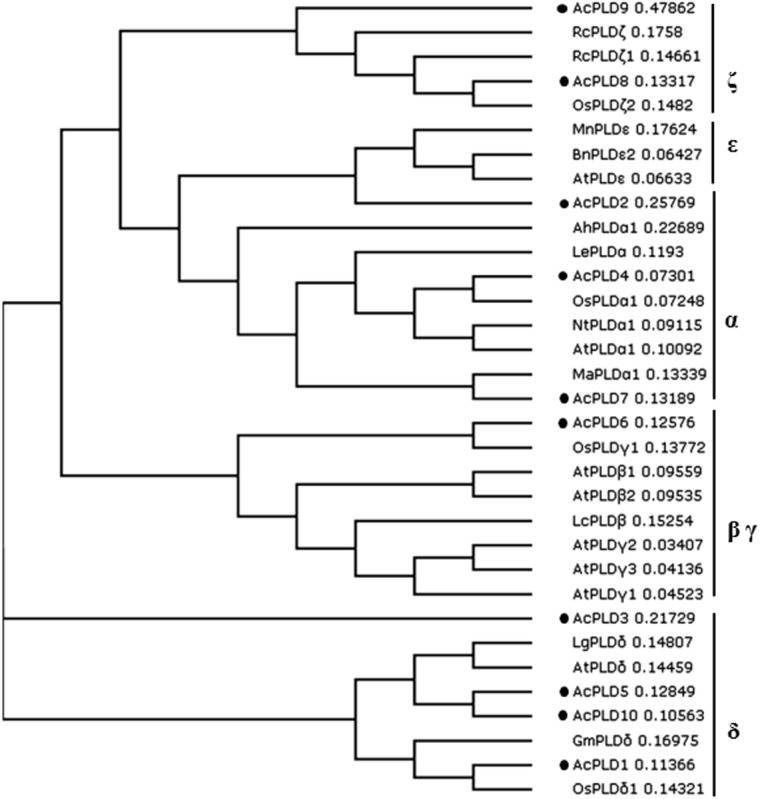
Phylogenetic tree illustrated the genetic relationships between 10 pineapple PLDs (marked with black solid circles) and the other PLDs of 23 species. Sequence name and GenBank accession number in the figure are shown as follows: LgPLD_δ_ (JF791814.1), OsPLD_αl_ (NM_001048688.1)_,_ NtPLD_α1_ (XM_009627397.1), LcPLD_β_ (HQ833032.1), AhPLD_α1_ (AB232323.1), MaPLD_α1_ (XM_009403448.1), AtPLD_α1_ (NM_112443.2), LePLD_α_ (NM_001302895.2)_,_ GmPLD_δ_ (XM_003532746.2), RcPLD_ζ_ (XM_002512424.1)_,_ AtPLDβ1 (NP_565963.2)_,_ AtPLDγ1 (NP_192922.1), AtPLDγ2 (NP_849539.1)_,_ BnPLD𝜀2 (XP_013643667.1), AtPLDγ3 (NP_192921.1)_,_ AtPLDδ (NP_849501.1), AtPLD𝜀 (NP_175914.1), AtPLDβ2 (NP_567160.1), OsPLDδ1 (XP_015647989.1), OsPLDζ2 (XP_015622124.1), OsPLDγ1 (XP_015614421.1). RcPLDζl (XP_015573381.1), MnPLD𝜀 (XP_010106671.1).

To examine the phylogenetic relationship between the AcPLD proteins and other plant species, a phylogenetic tree was constructed on the basis of their translated amino acid sequences. The whole plant PLD proteins could be grouped into six types: α, β, γ, δ, 𝜀, and ζ (Qin and Wang, 2002). Based on the tree, the 10 *PLD* genes in pineapple were clustered into five types. AcPLD2, AcPLD4, and AcPLD7 were clustered to α type, and AcPLD6 was clustered to β or γ type. AcPLD1, AcPLD3, AcPLD5, and AcPLD10 were δ type, whereas AcPLD8 and AcPLD9 were ζ type (**Figure 4**).

To investigate the potential function of the AcPLD proteins, the transmembrane helix number was analyzed via topology prediction using HMMTOP online tools. The resulting data showed that zero, one and two transmembranes existed in the associated AcPLDs (**Table 1**).

These data suggested that AcPLD1–AcPLD10 exhibited great structural diversity and possibly exerted different physiological functions.

### *PLD* Gene Expression in Pineapple during IB

To understand the possible role of *AcPLD1*–*AcPLD10* in pineapple during IB, the expression patterns of *AcPLD1*–*AcPLD10* in pulp of fruits treated with hexaldehyde were investigated by qRT-PCR. As shown in **Table 4**, among the 10 *AcPLD* genes, *AcPLD2* (1.6- to 5.7-fold) was strongly induced after hexaldehyde treatment. By contrast, the transcript level of *AcPLD9* (1.9- to 4.0-fold) decreased following hexaldehyde treatment. *AcPLD7* and *AcPLD8* showed similar expression patterns, and they decreased by 1.3- to 2.5-fold and 1.1- to 2.3-fold in treated fruits, respectively, and did not substantially change between the treated and non-treated fruits. In addition, *AcPLD1* and *AcPLD3* transcripts remained nearly unchanged, and *AcPLD4-6* fluctuated for both control and treated fruits during storage.

**Table 4:** qRT-PCR analysis of the 10 *PLD* genes in hexaldehyde-treated pineapple fruits during storage at 25°C for 12 days.

Name	Treatment	Storage days						
		0	2	4	6	8	10	12
*PLD1*	Control	1.00 ± 0.05	1.19 ± 0.04	0.54 ± 0.024	0.81 ± 0.065	0.48 ± 0.032	0.37 ± 0.021	0.42 ± 0.021*
	Hexaldehyde	1.00 ± 0.04	1.20 ± 0.032	0.59 ± 0.024	0.83 ± 0.025	0.46 ± 0.011	0.36 ± 0.021	0.57 ± 0.018
*PLD2*	Control	1.00 ± 0.37	0.91 ± 0.19*	0.77 ± 0.15*	0.79 ± 0.13*	1.32 ± 0.19*	2.00 ± 0.088*	1.98 ± 0.25*
	Hexaldehyde	1.00 ± 0.073	2.02 ± 0.32	2.59 ± 0.18	2.49 ± 0.15	3.19 ± 0.15	3.28 ± 0.15	11.29 ± 0.27
*PLD3*	Control	1.00 ± 0.05	1.12 ± 0.15	1.18 ± 0.099	0.94 ± 0.033	1.17 ± 0.018	0.58 ± 0.058	0.54 ± 0.21
	Hexaldehyde	1.00 ± 0.04	1.20 ± 0.06	1.09 ± 0.035	1.03 ± 0.078	1.11 ± 0.095	0.62 ± 0.027	0.57 ± 0.035
*PLD4*	Control	1.00 ± 0.039	0.63 ± 0.084	0.60 ± 0.021*	0.16 ± 0.038	0.17 ± 0.048	0.34 ± 0.015	0.35 ± 0.036
	Hexaldehyde	1.00 ± 0.06	0.22 ± 0.028*	0.73 ± 0.049	0.14 ± 0.021	0.18 ± 0.012	0.10 ± 0.046*	0.26 ± 0.013*
*PLD5*	Control	1.00 ± 0.021	0.92 ± 0.042	1.15 ± 0.01	1.13 ± 0.008	1.12 ± 0.007	0.74 ± 0.051*	0.72 ± 0.042
	Hexaldehyde	1.00 ± 0.034	0.88 ± 0.035	1.16 ± 0.008	1.03 ± 0.041*	1.06 ± 0.01	1.01 ± 0.006	0.78 ± 0.042
*PLD6*	Control	1.00 ± 0.15	1.80 ± 0.19*	3.89 ± 0.19	4.39 ± 0.28*	3.67 ± 0.15	4.55 ± 0.26	4.32 ± 0.44
	Hexaldehyde	1.00 ± 0.14	2.11 ± 0.16	2.63 ± 0.55*	5.78 ± 0.25	3.53 ± 0.16	1.64 ± 0.63*	2.89 ± 0.17*
*PLD7*	Control	1.00 ± 0.088	1.79 ± 0.12*	4.59 ± 0.34	4.28 ± 0.28	2.37 ± 0.15	1.97 ± 0.12	3.05 ± 0.27
	Hexaldehyde	1.00 ± 0.079	2.23 ± 0.18	1.86 ± 0.15*	3.19 ± 0.17*	2.49 ± 0.14	2.03 ± 0.13	2.42 ± 0.24*
*PLD8*	Control	1.00 ± 0.045	1.98 ± 0.086	3.87 ± 0.15	3.90 ± 0.16	3.12 ± 0.15	1.74 ± 0.15*	2.48 ± 0.16
	Hexaldehyde	1.00 ± 0.057	1.34 ± 0.086*	1.66 ± 0.15*	3.42 ± 0.20*	3.01 ± 0.19	3.48 ± 0.19	2.52 ± 0.096
*PLD9*	Control	1.00 ± 0.19	1.25 ± 0.049	2.05 ± 0.14	2.36 ± 0.038	1.89 ± 0.18	1.04 ± 0.16	1.60 ± 0.12
	Hexaldehyde	1.00 ± 0.054	0.49 ± 0.024*	0.51 ± 0.042*	0.67 ± 0.051*	0.80 ± 0.067*	0.55 ± 0.042*	0.53 ± 0.025*
*PLD10*	Control	1.00 ± 0.031	1.49 ± 0.061	2.07 ± 0.14	4.21 ± 0.15	1.33 ± 0.069*	1.88 ± 0.14	2.15 ± 0.12
	Hexaldehyde	1.00 ± 0.18	1.19 ± 0.14*	0.87 ± 0.038*	2.38 ± 0.087*	1.83 ± 0.041	1.65 ± 0.079	1.94 ± 0.057

*^∗^Significant difference between control and hexaldehyde treatment at the P ≤ 0.05 level by t-test.*

### Subcellular Localization of AcPLD2 and AcPLD9 Proteins

**FIGURE 5:**
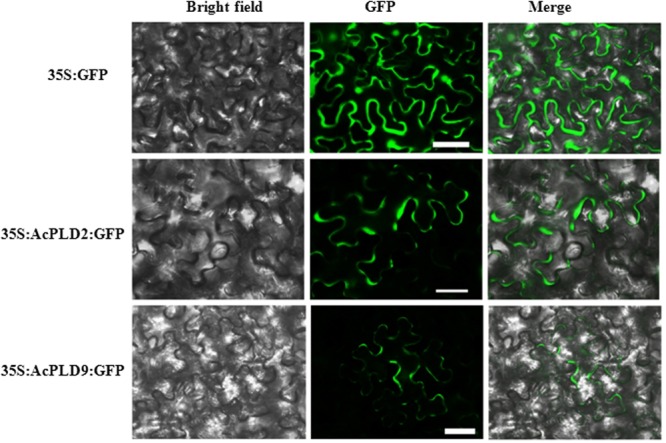
Subcellular localization of AcPLD2 andAcPLD9 in tobacco (*N. benthamiana*) leaf epidermal cells. Tobacco epidermal cells were transiently transformed with constructs containing either fusion plasmids (AcPLD2-GFP and AcPLD9-GFP) or control (GFP alone) via *Agrobacterium* inoculation, GFP fluorescence was detected 24 h after inoculation. Bar = 50 μrn.

AcPLD2 and AcPLD9 were selected for subcellular localization analysis due to their gene expression patterns with obvious characteristics during IB. To detect the potential role of the two proteins (AcPLD2 and AcPLD9), we assayed their localizations in the cell. The results indicated that the fluorescent signals from the AcPLD2-GFP and AcPLD9-GFP constructs were observed in the plasma membrane of tobacco leaf epidermal cells, whereas the fluorescence from the GFP control was distributed within the cells (**Figure 5**).

### Contents of H_2_O_2_ and Enzymes Related to H_2_O_2_

To further investigate the potential regulatory mechanism of hexaldehyde treatment alleviating IB, we analyzed the contents of H_2_O_2_ and enzymes related to H_2_O_2_. As shown in **Table 5**, the SOD content was decreased before the eighth day of storage and then increased in the treated fruits compared with the control samples. The contents of CAT, NADPH, and APX abundances showed similar patterns; they increased and peaked after 6 days of storage and then decreased in treated and non-treated fruits. However, the CAT, NADPH, and APX levels were suppressed at day 2, and their contents were induced in treated fruits than in non-treated samples. The H_2_O_2_ content clearly fluctuated after hexaldehyde treatment during storage, increased at days 4 and 6, decreased at day 8, and increased again at day 12 in treated fruits. A significant difference was observed between the treated and non-treated fruits.

**Table 5:** Changes in SOD, CAT, NADPH, APX, H_2_O_2_, and PLD contents (ng.mg^-1^protein) in hexaldehyde-treated pineapple fruits during storage at 25°C for 12 days.

Name	Treatment	Storage days						
		0	2	4	6	8	10	12
SOD	Control	0.557 ± 0.0012	0.389 ± 0.0053	0.307 ± 0.0069*	0.774 ± 0.019	0.602 ± 0.028	0.239 ± 0.0019*	0.223 ± 0.011*
	Hexaldehyde	0.557 ± 0.0012	0.346 ± 0.0039*	0.510 ± 0.021	0.497 ± 0.0049*	0.498 ± 0.0035*	0.854 ± 0.034	0.445 ± 0.015
CAT	Control	2.714 ± 0.108	3.735 ± 0.174	2.591 ± 0.0059	5.139 ± 0.076*	2.998 ± 0.188*	2.502 ± 0.088*	2.409 ± 0.309*
	Hexaldehyde	2.714 ± 0.108	1.633 ± 0.122*	2.373 ± 0.384	6.145 ± 0.073	3.891 ± 0.0068	4.059 ± 0.250	3.188 ± 0.336
NADPH	Control	0.338 ± 0.0287	0.326 ± 0.0133	0.207 ± 0.0032*	0.483 ± 0.0228	0.399 ± 0.0466	0.236 ± 0.0206*	0.159 ± 0.0044*
	Hexaldehyde	0.338 ± 0.0287	0.166 ± 0.0287*	0.398 ± 0.0223	0.494 ± 0.0509	0.417 ± 0.0117	0.369 ± 0.0367	0.292 ± 0.0376
APX	Control	8.404 ± 0.186	7.343 ± 0.0698	4.843 ± 0.0415*	11.452 ± 0.497	5.487 ± 0.585*	3.901 ± 0.259*	4.039 ± 0.258
	Hexaldehyde	8.404 ± 0.186	4.897 ± 0.182*	6.614 ± 0.426	10.750 ± 0.378	7.628 ± 0.259	7.607 ± 0.284	3.791 ± 0.186
H_2_O_2_	Control	0.0089 ± 0.0004	0.010 ± 0.00011	0.007 ± 0.0003*	0.010 ± 0.0063*	0.011 ± 0.00065	0.0099 ± 0.0005	0.005 ± 0.0002*
	Hexaldehyde	0.0089 ± 0.0004	0.0010 ± 0.0002	0.0099 ± 0.0001	0.013 ± 0.0044	0.008 ± 0.0005*	0.001 ± 0.0006	0.0082 ± 0.0004
PLD	Control	0.043 ± 0.00026	0.025 ± 0.00087	0.020 ± 0.0009*	0.047 ± 0.0023	0.041 ± 0.00019	0.030 ± 0.0005*	0.032 ± 0.0001*
	Hexaldehyde	0.043 ± 0.00026	0.026 ± 0.00021	0.044 ± 0.00019	0.046 ± 0.0068	0.037 ± 0.0007*	0.037 ± 0.0003	0.045 ± 0.00035

*^∗^Significant difference between control and hexaldehyde treatment at the P ≤ 0.05 level by t-test.*

### PLD Content

Hexaldehyde-treated fruits showed slightly higher PLD contents than control samples on day 2. However, on day 4, the PLD content was significantly higher in the former than in the latter. Hexaldehyde-treated fruits had lower PLD content than control samples, and treated fruits had relatively higher PLD content than non-treated fruits. Significant differences were noted between the groups (**Table 5**).

## Discussion

Phospholipase D is an important signaling enzyme that has been identified in various species. A total of 12, 17, 11, and 18 PLD members have been characterized in *Arabidopsis* (Qin and Wang, 2002), in rice (*Oryza sativa*) (Li et al., 2007), grape (*Vitis vinifera*), and poplar (*Populus trichocarpa*) (Liu et al., 2010) genomes, respectively. Genome-wide identification of *PLD* genes in pineapples has not been reported. The recent completion of pineapple genome sequencing allows us to identify all PLD protein coding genes in pineapples via homology analyses. In the present study, 10 putative proteins in the pineapple genome database exhibited significant sequence identity with PLD homolog upon comparison with the NCBI data bank. We performed comprehensive analyses of the 10 AcPLD proteins with 23 other species such as rice and *Arabidopsis* of PLD protein for reference. Phylogenetic analysis indicated that the 10 identified AcPLD proteins could be classified into five major orthologous types (α, β, γ, δ, and ζ). The results of our study were inconsistent with previous reports on rice (α, β, κ, δ, φ, and ζ) (Li et al., 2007) and *Arabidopsis* (α, β, γ, δ, 𝜀, and ζ) (Pinosa et al., 2013) based on sequence similarity, thereby indicating the evolution of PLDs in different species. Phylogenetic analysis also revealed the evolutionary relationships among the AcPLD proteins, and the δ type contained the largest number (4) of PLD protein family members.

The involvement of the *PLD* gene in various biological processes including seed germination (Li et al., 2007), cold acclimation (Kargiotidou et al., 2010), resistance against pathogens (Zhao et al., 2013), and cold stress responses (Margutti et al., 2017), has been reported. In this study, the *AcPLDs* exhibited differential expression patterns during IB of pineapple. Among the 10 *AcPLD* genes, *AcPLD6*, *AcPLD7*, *AcPLD8*, *AcPLD9*, and *AcPLD10* were induced prior to IB (6 days). Furthermore, *AcPLD9* was downregulated by hexaldehyde treatment, which implied that AcPLD9 protein functioned as a positive regulator during IB. Our results were similar to the findings a previous report on PLD in *Arabidopsis* that showed a positive effect on pathogen defense (Pinosa et al., 2013). Notably, *AcPLD2* was upregulated in the presence of hexaldehyde, thereby indicating that AcPLD2 protein could function as a repressor in response to hexaldehyde treatment in pineapple. Similarly, Yamaguchi et al. (2009) demonstrated that OsPLDβ1 negatively regulates resistance against pathogens. PLD proteins are suggested to act as a membrane protein (Pinosa et al., 2013) and most isolated PLD proteins are localized in the plasma membrane (Wang and Wang, 2001; Pinosa et al., 2013). Further subcellular localization analyses demonstrated that AcPLD2 and AcPLD9 proteins were localized in the membrane. Thus, the two proteins may function within the plasma membrane.

A growing number of studies have suggested that PLD plays vital roles in response to environmental stresses in plants (Li et al., 2004; Wang, 2005; Liu et al., 2013). Activated PLD can hydrolyze phosphatidylcholine to produce PA, which stimulates the NADPH oxidase complex to generate H_2_O_2_ (Stahelin et al., 2003). Additionally, positive feedback exists between PLD and H_2_O_2_ (Xiao et al., 2005). However, little is known about the relationship between PLD and H_2_O_2_ during IB of pineapple. In this study, we showed that the production patterns of PLD and H_2_O_2_ were similar. Further hexaldehyde treatment suppressed PLD and H_2_O_2_ production in pineapple during late storage. These similarities suggested that PLD may inhibit H_2_O_2_ production. Similarly, PLD activity could specifically suppress H_2_O_2_-induced apoptosis in rats (Lee et al., 2000) and cell death in *Arabidopsis* (Zhang et al., 2003). Stahelin et al. (2003) reported a similar pattern of NADPH and H_2_O_2_ changes, thereby suggesting that NADPH may be a key enzyme in the induction of production H_2_O_2_ production. In view of the present observations, we propose that hexaldehyde treatment resulted in decreased *PLD* gene expression and diminished PLD enzyme abundance, decreasing H_2_O_2_ generation. However, our findings require verification.

In summary, a total of 10 putative *PLD* gene family members were identified in the pineapple genome. The 10 *PLD* genes were characterized based on their sequence identity, gene structure, conserved domains, phylogenetic analysis and gene expression profiles during IB by qRT-PCR. Data from this current study will assist in identifying *AcPLD* genes and provide a foundation for further study on the biological functions of PLD proteins in pineapples.

## Author Contributions

KH carried out the experiment. LZ participated in the research design, analyzing the data and drafting the manuscript, and initiated the project, designed the research framework, coordinated the research and reviewed the manuscript. RZ, BH, KS, and ZJ participated in data analysis and supported technically. All authors read and approved the final manuscript.

## Conflict of Interest Statement

The authors declare that the research was conducted in the absence of any commercial or financial relationships that could be construed as a potential conflict of interest.

## Footnotes

1 http://clustalw.ddbj.nig.ac.jp/

2 http://smart.embl-heidelberg.de/

3 http://www.ncbi.nlm.nih.gov

4 http://www.ebi.ac.uk/Tools/msa/clustalo/

5 http://nbcr-222.ucsd.edu/meme.html

6 http://web.expasy.org/peptide_mass/

7 http://www.enzim.hu/hmmtop/
