# Supplementary material for: Identification and Characterization of Phospholipase D Genes Putatively Involved in Internal Browning of Pineapple during Postharvest Storage
Source: Front Plant Sci. 2017 Jun 19;8:913. doi: 10.3389/fpls.2017.00913 (PMC5474486; doi:10.3389/fpls.2017.00913)
Supplement: Supplementary file 1 [file Table_1.DOC]

**Supplemental file.1:** Primer used for quantitative real-time PCR analysis.

| **Name** | **Forward primer (5′–3′)** | **Reverse primer ((5′–3′)** |
| --- | --- | --- |
| *Acactin* | CTGGCCTACGTGGCACTTGACTT | CACTTCTGGGCAGCGGAACCTTT |
| *AcPLD1* | TAACTGGCACTCCTACATTTACCC | CCAACCCTCCCATCTTTCTCT |
| *AcPLD2* | GCAAGCATCTCACCCACATC | CCTGACTCCGACACCCGTAT |
| *AcPLD3* | TAGCAGTAGGACAAAGTGGGAAC | CCAGAATCAATAGAGCGGAAAA |
| *AcPLD4* | AAAGGCGGACCAAGGGA | GAGGGATGATGATGTCAGAGAGA |
| *AcPLD5* | CAAACAGAAGGTTGTGGGAACT | GCAAATGTGGGATTGTGGAAA |
| *AcPLD6* | TGCCGAATACTTACTTCCCTTT | GCCAACACTTTCCATGCTGAT |
| *AcPLD7* | GGTTCCCCACACATTCTTCC | CATAGTACCTACCGCTGCCTAAA |
| *AcPLD8* | TTGTGCTGCTCGTTTATCCC | CACTACATCAATCAGACCCTTGCTA |
| *AcPLD9* | TGAACAAGGATGGGTTGAGACA | CCAGTAAGCGACAAGGAGGTG |
| *AcPLD10* | TAAAGGTTGAAGCAGACGGC | GGTTAGTGTATCAGGGAGCGAAG |
