# Supplementary material for: Identification and Characterization of Phospholipase D Genes Putatively Involved in Internal Browning of Pineapple during Postharvest Storage
Source: Front Plant Sci. 2017 Jun 19;8:913. doi: 10.3389/fpls.2017.00913 (PMC5474486; doi:10.3389/fpls.2017.00913)
Supplement: Supplementary file 2 [file Table_2.DOC]

**Supplemental file.2:** Primer used for fusing GFP.

| **Name** | **Sequence (5′–3′)** | **Restriction site** |
| --- | --- | --- |
| *AcPLD2-GFPFor* | AggatccATGCCTGCAGGTCGACGATTAAGCAGTGGT | *BamH* I |
| *AcPLD2-GFPRev* | AaagcttAGTGGTGAGAATAGGAGGCAGAACTTCAGA | *Hind* III |
| *AcPLD9-GFPFor* | AggatccATGCCGGATCTCCCCCGCGTCCCCG | *BamH* I |
| *AcPLD9-GFPRev* | AaagcttTGCCGACAACTGCAATGGCTTAACT | *Hind* III |
